# Identification of m^6^A RNA methylation genes in *Oryza sativa* and expression profiling in response to different developmental and environmental stimuli

**DOI:** 10.1016/j.bbrep.2024.101677

**Published:** 2024-03-12

**Authors:** Mahbub Hasan, Zakia Sultana Nishat, Md. Soyib Hasan, Tanvir Hossain, Ajit Ghosh

**Affiliations:** Department of Biochemistry and Molecular Biology, Shahjalal University of Science and Technology, Sylhet 3114, Bangladesh

**Keywords:** Epitranscriptomic marker, RNA methylation, Rice, Genome-wide analysis, Transcript profiling, m^6^A

## Abstract

Eukaryotic messenger RNAs (mRNAs) transcend their predominant function of protein encoding by incorporating auxiliary components that ultimately contribute to their processing, transportation, translation, and decay. In doing so, additional layers of modifications are incorporated in mRNAs at post-transcriptional stage. Among them, N6-methyladenosine (m^6^A) is the most frequently found mRNA modification that plays crucial roles in plant development and stress response. In the overall mechanism of m^6^A methylation, key proteins classified based on their functions such as writers, readers, and erasers dynamically add, read, and subtract methyl groups respectively to deliver relevant functions in response to external stimuli. In this study, we identified 30 m^6^A regulatory genes (9 writers, 5 erasers, and 16 readers) in rice that encode 53 proteins (13 writers, 7 erasers, and 33 readers) where segmental duplication was found in one writer and four reader gene pairs. Reproductive cells such as sperm, anther and panicle showed high levels of expression for most of the m^6^A regulatory genes. Notably, writers like *OsMTA*, *OsMTD*, and *OsMTC* showed varied responses in different stress and infection contexts, with initial upregulation in response to early exposure followed by downregulation later. *OsALKBH9A*, a noteworthy eraser, displayed varied expression in response to different stresses at different time intervals, but upregulation in certain infections. Reader genes like *OsECT5*, *OsCPSF30-L3*, and *OsECT8* showed continuous upregulation in exertion of all kinds of stress relevant here. Conversely, other reader genes along with *OsECT11* and *OsCPSF30-L2* were observed to be consistently downregulated. The apparent correlation between the expression patterns of m^6^A regulatory genes and stress modulation pathways in this study underscores the need for additional research to unravel their intricate regulatory mechanisms that could ultimately contribute to the substantial development of enhanced stress tolerance in rice through mRNA modification.

## Highlights

• N6-methyladenosine (m^6^A) is the most frequently found mRNA modification that plays crucial roles in plant development and stress response.
• We identified 30 m^6^A regulatory genes (9 writers, 5 erasers, and 16 readers) in rice that encode 53 proteins (13 writers, 7 erasers, and 33 readers).
• Segmental duplication was found in one writer and four reader gene pairs.
• High levels of expression for most of the m^6^A regulatory genes are observed in seed.

## 1 Introduction

Post-transcriptional RNA modifications or "epitranscriptomic" regulation have gained importance in recent years like epigenetic modifications in DNA [1,2]. Among all kingdoms of life, mRNAs, rRNAs, and tRNAs are decorated with more than 150 distinct chemical modifications that differ in degree, topology, and type [3]. In the case of tRNA, modifications occur in approximately 17% of total nucleotides whereas this amount is only 2% for rRNA [4]. Though the processes of mRNA modifications and their functions are still in emerging condition, mRNA modification is found to be less common in comparison to rRNA and tRNA [5]. Only a handful of different methylations [e.g. m^3^C, m^1^G, m^1^A, m^5^C, m^5^A, m^6^A, etc.] have been identified so far in mRNAs, where N6-methyladenosine (m^6^A) is the most abundant mRNA modification in both animals and plants [6]. In accomplishing this modification, three protein groups act as m^6^A methylation regulators, where each group has distinct functions. RNA methyltransferase (MT), also known as the "writer", and RNA demethylase (DMT), often named as "eraser" are two crucial protein groups that regulate the amounts and alterations of RNA methylation. A third protein group known as the "reader" is involved in the detection and processing of methylated mRNAs. In other words, writer, reader, and eraser protein groups are for installing, reading, and reversibly uninstalling methyl groups respectively (Fig. 1) onto the sixth nitrogen atom of the adenosine base of mRNA [7,8].

**Fig. 1:**
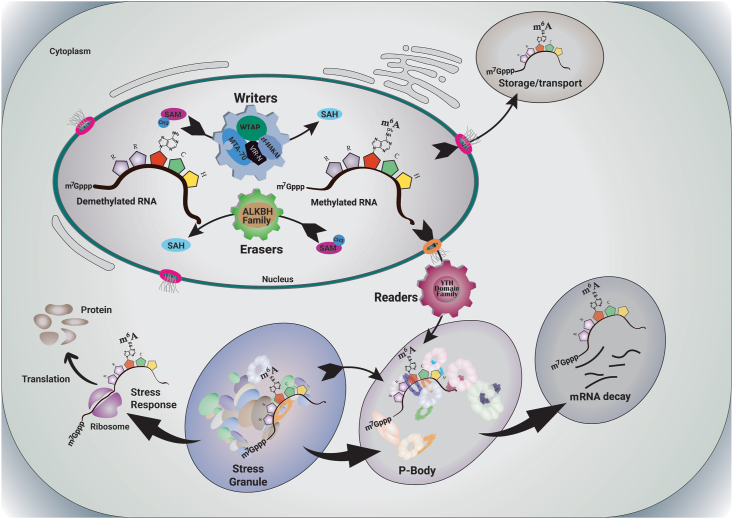
**Graphical presentation of plants' m**^**6**^**A regulatory genes and their functionality.** Writers of m^6^A are characterized by MTA-70, WTAP, VIR-N, and zf-HAKAI domains are responsible for the RNA methylation at the RRACH region. ALKBH domain is a distinctive trait of erasers, which are accountable for the demethylation of methylated RNAs. Lastly, the YTH domain family, which belongs to the group of m^6^A readers, is responsible for converting methylation marks into effector functions.

In mRNA, RRACH (R = Guanine or Adenine; H = Adenine, Cytosine or Uracil) is the most renowned specific consensus motif that is recognized by the methyltransferase “writer” proteins of the m^6^A pathway and methylates the RNAs [9]. Modern techniques like high-throughput m^6^A-seq, methylated RNA immunoprecipitation sequencing (MeRIP-seq), and methylation individual-nucleotide-resolution crosslinking and immunoprecipitation (miCLIP) have recently made significant advances in identifying other writer binding motifs in mRNA. The writer proteins contain several conserved domains that are useful in the identification of the respective protein's functional identity [8,10,11]. In plants, such domains are found in writer complexes like methyltransferase including MTA (ortholog of METTL-3), MTB (ortholog of METTL-14), FKBP12 interacting protein 37 kDa, FIP37 (ortholog of WTAP), VIR (ortholog of VIRMA) [9,[12], [13], [14]]. During the installation of a methyl group in mRNA, writer proteins transfer a methyl group from a donor by following different mechanisms. For example, METTL3 and METTL14 are two S-Adenosyl Methionine (SAM) dependent methyltransferase (SAM MTase) superfamily proteins that form a heterodimer catalytic core and transfer the methyl group of SAM to the adenosine of the target mRNA [15]. Besides, WTAP and VIRMA help to localize the catalytic core to nucleus speckles and recruit other N6-methyltransferase components to facilitate the process of writing [16,17]. Although there is no ortholog of Zc3h13 found in plants, E3 ubiquitin ligase (HAKAI) is reported as a component of the writer complex [[18], [19], [20]].

As installation of methyl group results in biological consequences, uninstallation has also its part in plant physiological processes. In uninstalling as well as removing the methyl groups from mRNA, eraser proteins carry out this process with their intrinsic enzymatic ability. In mammals, out of nine AlkB (Alkylation B) family proteins, only heme and α-ketoglutarate-dependent dioxygenase FTO (Fat mass and obesity protein) and nonheme and α-ketoglutarate-dependent dioxygenase ALKBH5 are counted as erasers for their functioning as RRACH motif specific m^6^A demethylase [[21], [22], [23], [24]]. On the other hand, ALKBH9B and ALKBH10B function as erasers in plants and several homologs of the ALKB family members are also found [25,26].

In the interval of dynamic action of writers and erasers, readers perform various functions such as regulating the stability, exportation, and translation of methylated RNAs. The YTH (YT521-B homology) domain-containing protein family (YTHDF) is a group of evolutionarily conserved m^6^A readers in which the YTF domain binds with RNA in an m^6^A-dependent manner [[27], [28], [29]]. In the subsequent stages different proteins act in different action such as HNRNP family proteins aid in RNA processing, IGF2BP 1–3 enhances stability, YTHDC1 partners with SRSF3 and NXF1 for N6-methylated mRNA export, YTHDF3 associates with EIF4G2 for circular RNA translation and YTHDF1 recruits EIF3 to form stress granule mRNA-protein loops [[30], [31], [32], [33], [34], [35]]. Evolutionarily conserved C terminal (ECT) region of the YTH domain and 30 kDa subunit of cleavage and polyadenylation specificity factor (CPSF30L) are two plant orthologs of readers among many others [[36], [37], [38]].

In the case of plant lifecycle, there has been growing evidence that dynamic patterning of m^6^A methylome is involved in all stages including development timing and morphogenesis [20,39,40]. A significant one worth mentioning here is the cellular arrest at the globular stage of embryo development when the writer gene MTA is knocked out from Arabidopsis [18]. Eraser enzyme modification also plays a crucial role. The magnitudes of the writer and eraser enzymes’ activity are also evident in different developmental anomalies. For example, reduced apical dominance, and a decrease in floral organ number and size are observed with the reduction of m^6^A level [41]. This reduction of m^6^A level also known as hypomethylation caused by overexpression of ALKBH10B also shows an early flowering phenotype whereas ALKBH10B mutant delays flowering and represses vegetative growth [25,41].

In a previous study, 29 m^6^A genes were identified in Arabidopsis, encompassing 55 proteins, consisting of 8 writer, 14 eraser, and 33 reader genes [42]. Recent researches, including reviews, have explored the significant impact of epitranscriptomic RNA modifications on post-transcriptional gene regulation and various physiological processes in plants, particularly during growth and responses to abiotic stresses [43]. As far as our current knowledge goes, comprehensive genome-wide studies specific to *Oryza sativa* remain scarce. Yet, rice holds immense significance as a staple food globally, contributing to approximately 20% of human calorie intake. Each year, over 600 million tons of rice are harvested from around 153 million hectares of land which faces ongoing threats due to climate change, including global warming-induced stresses [44]. As there exists a direct correlation between m^6^A-mediated transcriptomics and stress tolerance, it presents an intriguing avenue for research in addressing future challenges in the upcoming days. In the present study, a genome-wide identification was conducted in the monocot model plant *Oryza sativa* to identify writers, erasers, and readers genes of the m^6^A pathway. The expression profile of all the identified members was analyzed in various anatomical tissues, developmental stages, and in response to several unfavourable conditions.

## 2 Method and materials

### 2.1 Identification and nomenclature of mRNA m^6^A writer, eraser, and reader genes in *Oryza sativa*

To identify putative rice mRNA writer (methyltransferase) proteins, the BLASTP search in Phytozome (https://phytozome.jgi.doe.gov/pz/portal.html), [45] was conducted according to the previous report [42] using Arabidopsis thaliana m^6^-adenosine methyltransferase (NCBI ref. seq: NP_192814.1), E3-ubiquitin ligase Hakai (NP_195736.1), VIRILIZER (NP_001319481.1), FIP37 (NP_190985.1) protein sequences as the query. By using RNA demethylase ALKBH9A (NP_001031159.1) and ECT1 (NP_001030629.1) protein sequences as a query, the putative eraser (demethylase) and reader (YTH domain) proteins were identified from the same database, respectively. To ensure that neither of the putative identified members of the gene families left deserted, each of the identified protein sequences was further subjected to a secondary search with the Rice Genome Annotation Project database (http://rice.uga.edu/analyses_search_blast.shtml). Putative gene and corresponding protein sequences were retrieved from the database and the presence of conserved domains was confirmed by NCBI Conserved Domain (https://www.ncbi.nlm.nih.gov/Structure/cdd/wrpsb.cgi) and Pfam (http://pfam.xfam.org/). The identity of the “writer” family was confirmed by the presence of the MT-A70 domain (PF05063), Wtap domain (PF17098), Vir-N domain (PF15912), and zf-Hakai (PF18408). The presence of 2-OG Fe (II) oxygenase superfamily (PF13532) and YTH domain (PF04146) confirmed the identity of the “eraser” and “reader” families, respectively. The prefix "Os" for *Oryza sativa* (Rice) was added to all members followed by subclass IDs (MT, FIP37, VIRILIZER, HAKAI, ECT, CPSF, and ALKBH), and a number according to their chromosomal appearance.

From the Rice Genome Annotation Project Database (http://rice.uga.edu/index.shtml), different information such as chromosomal position, strand position, CDS coordinate (5′ to 3′), gene length, CDS length, and protein length were obtained. The ExPASy-ProtParam tool (https://web.expasy.org/protparam/) was used to determine the physicochemical characteristics of the discovered proteins, such as their theoretical isoelectric point and molecular weight [46]. Using the CELLO v.2.5 (http://cello.life.nctu.edu.tw/) and pSORT (http://www.genscript.com/wolf-psort.html) database, protein subcellular localization was predicted.

### 2.2 Chromosomal localization and gene duplication

Chromosomal location information was obtained from the Phytozome database (https://phytozome.jgi.doe.gov/pz/portal.html) for all identified writers, erasers, and readers. Plant Genome Duplication Database (PGDD, http://pdgd.njau.edu.cn:8080/) was used to identify paralogous genes by counting duplication events between rice genes [47]. The same database was used to derive the rates of synonymous (Ks) and non-synonymous substitution (Ka). The Ka/Ks ratio was used to determine the selection pressure on duplicated genes. Consider as purifying selection when a pair of genes with Ka/Ks < 1, neutral drifting when a pair of genes with Ka/Ks = 1, and positive or Darwinian selection with Ka/Ks > 1. T = Ks/2λ was used to determine the approximate age (Mya, million years ago) of each duplication event [48]. The divergence time for monocots (a group of flowering plants with one cotyledon or seed leaf) is about 130 million years ago (Mya), and the synonymous substitution rate for grasses is about 6.5 x 10^−9^ substitutions per site per year [49,50]. Chromosomal distribution of the m^6^A gene and gene duplication data is plotted with Circos software (http://circos.ca/) [51].

### 2.3 Multiple sequence alignment and phylogenetic analysis

All the writer, eraser, and reader proteins from 20 species including Red Algae: *Porphyra umbilicalis*, Green Algae: *Chlamydomonas reinhardtii*, Moss: *Ceratopteris richardii*, Dicots: *Amborella trichopoda*, *Aquilegia coerulea*, *Glycine max, Arabidopsis thaliana*, *Coffea arabica, Solanum lycopersicum, Linum usitatissimum*, *Gossypium barbadense, Citrus clementina, Corymbia citriodora*, and *Anacardium occidentale,* Monocots: *Zea mays, Triticum aestivum, Oryza sativa, Panicum hallii, Brachypodium hybridum* and *Acorus americanus* were collected from Phytozome database (https://phytozome.jgi.doe.gov/pz/portal.html). Using the MUSCLE algorithm, multiple sequence alignment was carried out [52]. Molecular Evolutionary Genetics Analysis (MEGA)-X software was used to create the phylogenetic trees of the writer, eraser, and reader families [53]. A neighbour-joining tree technique was used in conjunction with bootstrap tests with 1000 replicates to evaluate the statistical reliability of each node. The Jones-Taylor-Thorton (JTT) model was used to do the neighbour-joining analysis, and the pairwise deletion option with a 95% site coverage criterion was used for the handling of gaps or missing data. The Nearest-Neighbour-Interchange (NNI) ML heuristic approach was chosen for tree inference.

### 2.4 Gene structure and domain architecture of writer, eraser, and reader proteins

Using Gene Structure Display Server 2.0 (http://gsds.gao-lab.org/), the physical mapping of the exon-intron sequence has been projected on a graph by comparing the genomic sequence and their matching coding DNA sequence obtained from the Phytozome database [54]. The position of the exon-intron and upstream-downstream sequences were included in the result.

To determine the occupancy of conserved MT-A70, Wtap, Vir-N, and Zf-Hakai domains for mRNA m^6^A writers, 2-OG-Fe (II) oxygenase domain for erasers, and YTH domain for readers, all the identified writer, eraser, and reader proteins were evaluated using the Pfam database [55]. Comparative domain location has been graphically shown using the starting and ending positions of the detected domains for the constituent amino acids.

### 2.5 3D structure prediction of m^6^A proteins

AlphaFold2 structure prediction tool was utilized to create comprehensive 3D models of the writer, reader, and eraser proteins. AlphaFold2 employs a neural network method to generate protein structures with remarkable accuracy and reliability, which has revolutionized protein structure prediction by achieving experimentally similar or nearly accurate resolutions [56]. Protein sequences acquired earlier from the Phytozome database were employed for predicting 3D structures. The structure of these proteins was analyzed using the PyMol visualization tool.

### 2.6 Expression profiling of RNA writer, eraser, and reader genes at different tissues and developmental stages

Rice RNA writer, eraser, and reader genes RNA-Seq data were obtained from the Rice Expression Database (RED) (http://expression.ic4r.org/) [57]. The RED serves as a comprehensive repository of gene expression profiles, exclusively derived from RNA-Seq data analysis. RED integrates and visualizes these gene expression profiles, ensuring they are based on meticulously curated and quality-controlled RNA-Seq data. Rice tissues are included in these transcript data, including anther, callus, aleurone, root, seed, panicle, leaf, pistil, and shoot. From the boxplot data of 284 expression profiling results of a gene, the mean value of the different tissue-specific expression levels of the 30 m^6^A genes was carried out to generate a heatmap using the GraphPad prism tool [58,59].

### 2.7 Expression profiling in response to diverse abiotic and biotic stresses

The normalized and curated fold change in expression data for the rice RNA writer, eraser, and reader genes were obtained from the Genevestigator database (https://genevestigator.com/), with default settings using experiment IDs for abiotic stresses (OS-00102 for cold, OS-00287 for salinity, OS-00232 for OS-00225 for dehydration) and biotic stresses (OS-00034 for *X. oryzae pv. oryzae*, OS-00045 and OS-00285 for *M. oryzae*, OS-00011 for *M. grisea*, OS-00079 for *M. graminicola*, and OS-00074 for *A. tumefaciens*). There were four abiotic stresses including cold, salt, wounding, and drought; and five pathogens: *X. oryzae pv. oryzae, M. oryzae, M. grisea, M. graminicola,* and *A. tumefaciens* infection with different time points. Heatmaps with hierarchical clustering were generated using the MeV program [59].

### 2.8 Identification of *cis*-regulatory elements in the putative promoter

The 1000 bp 5′ upstream sequences for all the writer, eraser, and reader genes were obtained from the Phytozome Database and scanned using the PlantCare Database (http://bioinformatics.psb.ugent.be/webtools/plantcare/html/) to determine the presence of significant stress-responsive *cis*-regulatory elements [60]. Schematic representations show the presence of identified *cis*-acting elements on each gene's putative promoter region.

## 3 Results

### 3.1 Characterization of the identified rice RNA m^6^A writer, eraser, and reader

We have identified a total of 13 writers, 7 erasers, and 33 reader proteins (a total of 53) in rice, which are encoded by nine writers, five erasers, and 16 reader genes (a total of 30). In all three families, there are more proteins than genes, indicating that RNA m^6^A altering transcripts had undergone alternative splicing. The length of transcripts, coding DNA sequence (CDS), polypeptide length, molecular weight, isoelectric point, and sub-cellular localization of each member of the newly identified writer, eraser, and reader families were further analyzed (Table 1). The CDS lengths of the rice genome writers range from 1059 bp to 6387 bp. Therefore, *OsVIRILIZER*, with its polypeptide length of 2128 amino acids and molecular weight of 233.746 kDa, is the largest member of the writing family, whereas *OsFIP37*, with its polypeptide length of 352 amino acids and molecular weight of 39.357 kDa, is the smallest member. Writers in the rice family had pI values ranging from 5.11 (*OsFIP37*) to 9.11 (*OsMTF*). Six of the eight writers had an acidic pI (below 7.0), whereas the other two were alkaline (basic). All the rice writers were predicted to be nuclear-localized, suggesting that they contribute to the methylation of the transcriptome.

**Table 1:** List of identified RNA m^6^A genes in rice along with their detailed information and localization.

Sl no		Gene Name	Locus Name	Transcripts	Coordinate (5’ - 3′)	Strand	Length (bp)		Protein			Localization
							Gene	CDS	Length	MW (kDa)	pI	
		*OsMTA*	LOC_Os01g16180	LOC_Os01g16180.1 LOC_Os01g16180.2 LOC_Os01g16180.3 LOC_Os01g16180.4	9159606–9165693	+	6088	2295	764	85.299	6.75	Nu^a^^,^^b^
mRNA m^6^A writer	1	*OsMTB*	LOC_Os02g45110	LOC_Os02g45110.1	27360417–27365800	+	5384	2121	706	77.803	6.76	Nu^a^^,^^b^, Mt^a^, Cy^b^
	2	*OsMTC*	LOC_Os03g05420	LOC_Os03g05420.1 LOC_Os03g05420.2	2676953–2682146	–	5194	2262	753	83.591	6.75	Nu^a^^,^^b^, Cy^b^
	3	*OsMTD*	LOC_Os10g31030	LOC_Os10g31030.1	16201250–16208251	+	7002	3042	1013	113.61	6.17	Nu^a^^,^^b^
	4	*OsMTE*	LOC_Os03g10224	LOC_Os03g10224.1	5202705–5206440	–	3736	1284	427	49.392	8.49	Nu^a^^,^^b^, Cy^b^
	5	*OsMTF*	LOC_Os03g10220	LOC_Os03g10220.1	5200523–5206413	–	5891	2214	737	83.337	9.11	Nu^a^^,^^b^
	6	*OsFIP37*	LOC_Os06g27970	LOC_Os06g27970.1	15863214–15872704	–	9491	1059	352	39.357	5.11	Nu^a^
	7	*OsVIRILIZER*	LOC_Os03g35340	LOC_Os03g35340.1	19585427–19604619	–	19193	6387	2128	233.746	5.16	Nu^a^^,^^b^
	8	*OsHAKAI*	LOC_Os10g35190.1	LOC_Os10g35190.1	18794537–18798562	+	4026	1509	502	53.121	6.70	Nu^a^^,^^b^
	9	*OsALKBH8A*	LOC_Os10g02760	LOC_Os10g02760.1 LOC_Os10g02760.2	1089548–1085169	–	4380	1791	597	62.844	8.60	Nu^a^,Cp^b^, Cy^b^,Go^b^
mRNA m^6^A Eraser	10	*OsALKBH8B*	LOC_Os03g13560	LOC_Os03g13560.1	7335053–7329103	–	5951	1977	659	69.993	8.64	Nu^a^^,^^b^, Cp^b^,Cy^b^
	11	*OsALKBH9A*	LOC_Os06g04660	LOC_Os06g04660.1 LOC_Os06g04660.2	2030467–2034813	+	4347	1851	616	68.073	6.08	Nu^a^^,^^b^, Cy^b^
	12	*OsALKBH9B*	LOC_Os11g29690	LOC_Os11g29690.1	17208164–17210164	+	2001	402	133	14.572	4.57	Nu^b^, Cy^b^
	13	*OsALKBH9C*	LOC_Os05g33310	LOC_Os05g33310.1	19528980–19525642	–	3339	1113	371	40.712	10.30	Cp^b^,Pm^a^,Mt^a^,Cy^a^^,^^b^
	14	*OsECT1*	LOC_Os01g22630	LOC_Os01g22630.1 LOC_Os01g22630.2	12725141–12731526	+	6386	2127	708	78.205	6.86	Nu^a^^,^^b^
mRNA m^6^A reader	15	*OsECT2*	LOC_Os01g48790	LOC_Os01g48790.1 LOC_Os01g48790.2 LOC_Os01g48790.3	27983688–27990383	+	6696	1830	609	67.071	5.65	Nu^a^^,^^b^
	16	*OsECT3*	LOC_Os03g06240	LOC_Os03g06240.1 LOC_Os03g06240.2	3125933–3130483	–	4551	2127	708	76.575	8.10	Nu^a^^,^^b^
	17	*OsECT4*	LOC_Os03g20180	LOC_Os03g20180.1 LOC_Os03g20180.2	11402649–11408108	+	5458	2130	709	77.046	5.50	Nu^a^^,^^b^
	18	*OsECT5*	LOC_Os08g44200	LOC_Os08g44200.1	27825032–27830316	–	5285	1875	624	68.266	5.13	Nu^a^^,^^b^
	19	*OsECT6*	LOC_Os03g53670	LOC_Os03g53670.1 LOC_Os03g53670.2	30777720–30781832	+	4113	1986	661	71.75	8.35	Nu^a^^,^^b^
	20	*OsECT7*	LOC_Os04g04000	LOC_Os04g04000.1 LOC_Os04g04000.2	1843006–1848258	+	5253	2028	675	76.439	6.08	Nu^a^^,^^b^
	21	*OsECT8*	LOC_Os04g51940	LOC_Os04g51940.1 LOC_Os04g51940.2 LOC_Os04g51940.3	30821134–30825520	+	4387	1725	574	63.793	5.58	Nu^a^^,^^b^
	22	*OsECT9*	LOC_Os05g01520	LOC_Os05g01520.1	304131–311132	–	7002	1917	638	73.169	6.90	Nu^a^^,^^b^
	23	*OsECT10*	LOC_Os05g06740	LOC_Os05g06740.1	3515604–3518444	–	2841	1221	406	44.067	9.11	Nu^a^
	24	*OsECT11*	LOC_Os07g07490	LOC_Os07g07490.1 LOC_Os07g07490.2	3726574–3731170	–	4597	1809	602	66.242	7.61	Nu^a^^,^^b^
	25	*OsECT12*	LOC_Os08g12760	LOC_Os08g12760.1 LOC_Os08g12760.2 LOC_Os08g12760.3 LOC_Os08g12760.4	7559098–7563188	–	4091	1734	577	63.867	5.41	Nu^a^^,^^b^
	26	*OsCPSF30-L1*	LOC_Os01g15300	LOC_Os01g15300.1 LOC_Os01g15300.2	8560623–8569631	–	9009	3024	1007	113.227	6.66	Nu^a^^,^^b^, Cy^b^
	27	*OsCPSF30-L2*	LOC_Os01g39100	LOC_Os01g39100.1	21973687–21980482	+	6795	2355	784	84.584	5.71	Nu^a^^,^^b^
	28	*OsCPSF30-L3*	LOC_Os06g21390	LOC_Os06g21390.1 LOC_Os06g21390.2 LOC_Os06g21390.3 LOC_Os06g21390.4	12352310–12358104	+	5795	1107	368	41.673	7.96	Nu^a^^,^^b^, Cy^b^
	29	*OsCPSF30-L4*	LOC_Os06g46400	LOC_Os06g46400.1	28151360–28156784	+	5425	1998	665	72.598	6.32	Nu^a^^,^^b^, Cy^b^
	30											

Abbreviations: CDS, coding DNA Sequence; PP, Polypeptide; MW, Molecular Weight; pI, Isoelectric point; bp, base pair; aa, amino acid; kDa, kilodalton; Cp, Chloroplast; Ec, Extracellular; Cy, Cytoplasm; Mt, Mitochondria; Nu, Nucleus; *Pm*, Plasma-membrane.

a Localization prediction by CELLO v.2.5 (http://cello.life.nctu.edu.tw/).

b Localization prediction by pSORT (http://www.genscript.com/wolf-psort.html).

Five Rice erasers were found to have CDS lengths ranging from 402 bp to 1977 bp, respectively. *OsALKBH8B* is 659 amino acids in length and has a molecular weight of 69.993 kDa, making it the longest and largest eraser protein. *OsALKBH9B* is the smallest eraser protein, with a molecular weight of 14.572 kDa. The pI values for both *OsALKBH9A* and *OsALKBH9B* erasers are acidic, and the rest of the erasers are basic. All erasers are mainly nuclear, as predicted by their subcellular location. Similarly, a wide range in CDS length, from 1830 base pairs (bp) to 2130 bp (bp) was observed among 16 reader genes. Accordingly, *OsECT2* is the shortest reader protein 609 aa long with a weight of 67.071 kDa, whereas *OsECT4* is the longest with 709 aa and 77.046 kDa. All except two rice readers are nuclear localized, just like the preceding two families (Table 1).

### 3.2 Chromosomal distribution and gene duplication

**Fig. 2:**
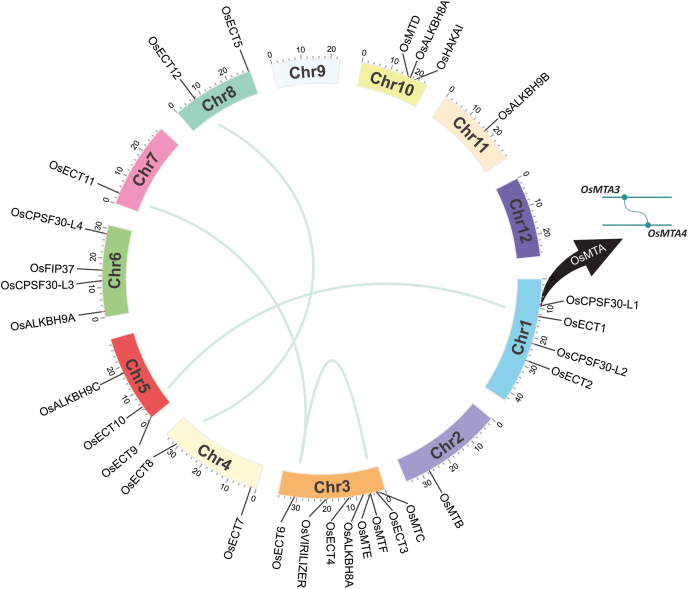
**Chromosomal distribution and gene duplication analysis of m**^**6**^**A genes.** 30 putative genes are unevenly distributed through the 12 rice chromosomes and duplicated gene information has been demonstrated using the CIRCOS visualization tool.

To explore the location of these genes on chromosomes we found that the identified putative 30 genes have been unevenly distributed throughout the seven chromosomes of rice. Eight of the total 30 genes are on chromosome 3, five on chromosome 1, four on chromosome 6, three each on chromosomes 5, and 10; two each on chromosomes 4, and 8; and one each on chromosomes 2, 7, and 11 (Fig. 2). The process of gene duplication and subsequent divergence is responsible for the evolution of plants (Fig. 2). The process of gene duplication and subsequent divergence is responsible for the evolution of plants [61,62]. The divergence between the two most important angiosperm groups, the eudicots, and the monocots, occurred between 125-140 Mya and 170–235 Mya ago, resulting in chromosomal duplication and gain/loss of genes [63]. One writer (*OsMTA3- OsMTA4*) and four readers gene pairs (*OsECT1-OsECT10, OsECT3-OsECT6, OsECT6- OsECT11*, and *OsECT8- OsECT12*) were found to be duplicated (Fig. 2 and Table 2). Among these genes, *OsMTA3* and *OsMTA4* were adjacent on chr1, which may have been caused by a tandem duplication event and the rest of the 4 duplications were due to segmental duplication. The proportion of non-synonymous to synonymous substitution (Ka/Ks) reveals the course of evolution and the nature of selection [64]. The Ka/Ks ratio for all these five gene pairings was below 1, indicating the role of purifying selection and the divergent times, ranging from 71.9 to 182.3 Mya.

**Table 2:** List of duplicated RNA m^6^A genes identified in rice.

Sl no	Locus1	Locus2	Ka	Ks	ka/ks	Type of duplication	MYA	Purifying selection
1	OsMTA3	OsMTA4	0.191	0.935	0.204	Segmental	46.75	Yes
2	OsECT1	OsECT10	1.0678	2.3703	0.45	Segmental	79.01	Yes
3	OsECT3	OsECT6	0.3443	1.683	0.2045	Segmental	56.1	Yes
4	OsECT6	OsECT11	0.4807	2.1889	0.2196	Segmental	72.96	Yes
5	OsECT8	OsECT12	0.1906	1.1497	0.1657	Segmental	39.69	Yes

### 3.3 Study of gene structure and conserved domains

**Fig. 3:**
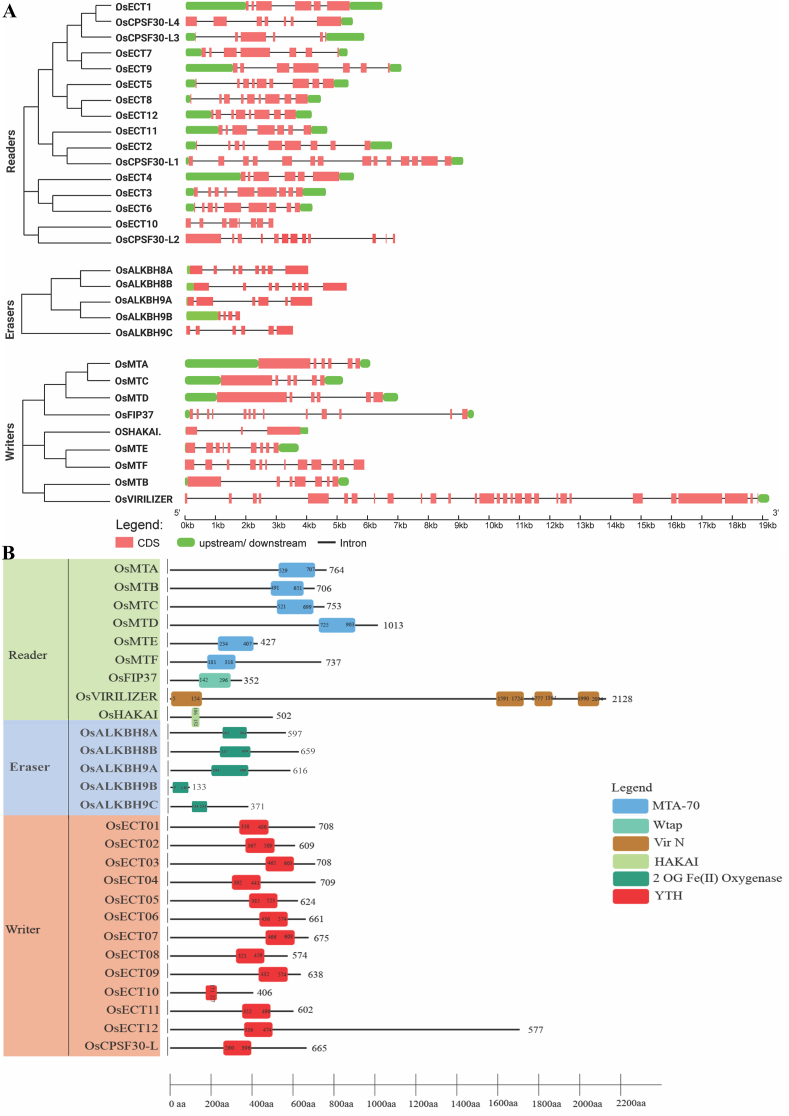
**Gene structure of m**^**6**^**A regulatory genes along with evolutionary relationship and their corresponding domain architecture.** (A) The relationship between the three groups was determined using MEGA X, resulting in a phylogenetic tree. GSDS was employed to generate diagrams exhibiting exon-intron sequences, with proportional length representation. The red color denotes exon segments while black lines represent introns. (B) Domain architectures were depicted based on the domain data retrieved from the Pfam database. The YTH reader domains were marked in red, while the Eraser domain 2OG-Fe (II) domains were indicated by a sap green colour. The MTA-70, WTAP, VIR-N, and zf-HAKAI writer domains were visually distinguished by dark blue, paste, brown, and olive colours.

The gene structure showed the presence of 3–27 exons for writers, 4–6 for erasers, and 4–14 for readers (Fig. 3A). Erasers have the fewest exons on average among the three families. *OsVIRILIZER* has the most exons among writers of 30, while *OsHAKAI* has the lowest of 3. Exon counts among readers range from 4 for *OsCPSF30-L3* to 14 for *OsCPSF30-L1*. A search of the Pfam database for the existence of conserved domains in proteins that are characteristic of certain populations but not others. In the writer family, MT-A70, Wtap, VIRN, and HAKAI are conserved domains. The MT-A70 domain is present in *OsMTA, OsMTB, OsMTC, OsMTC, OsMTD, OsMTE*, and *OsMTF* (Fig. 3B). Each of *OsFIP37, OsVIRILIZER*, and *OsHAKAI* is equipped with its unique domain: Wtap, VIRN, and HAKAI. The presence of the conserved domain 2-oxyglutarate and Fe (II) dependent oxygenase superfamily identifies RNA m^6^A erasers. All members of the eraser family have a conserved domain for 2-OGFe (II) oxygenase. Reader proteins were given the designation YTH (YT-521-B-like domain). All 13 rice reader proteins have the YTH domain, according to analyses by Pfam (Fig. 3B)

### 3.4 3D structures of the m^6^A proteins and their domain analysis

**Fig. 4:**
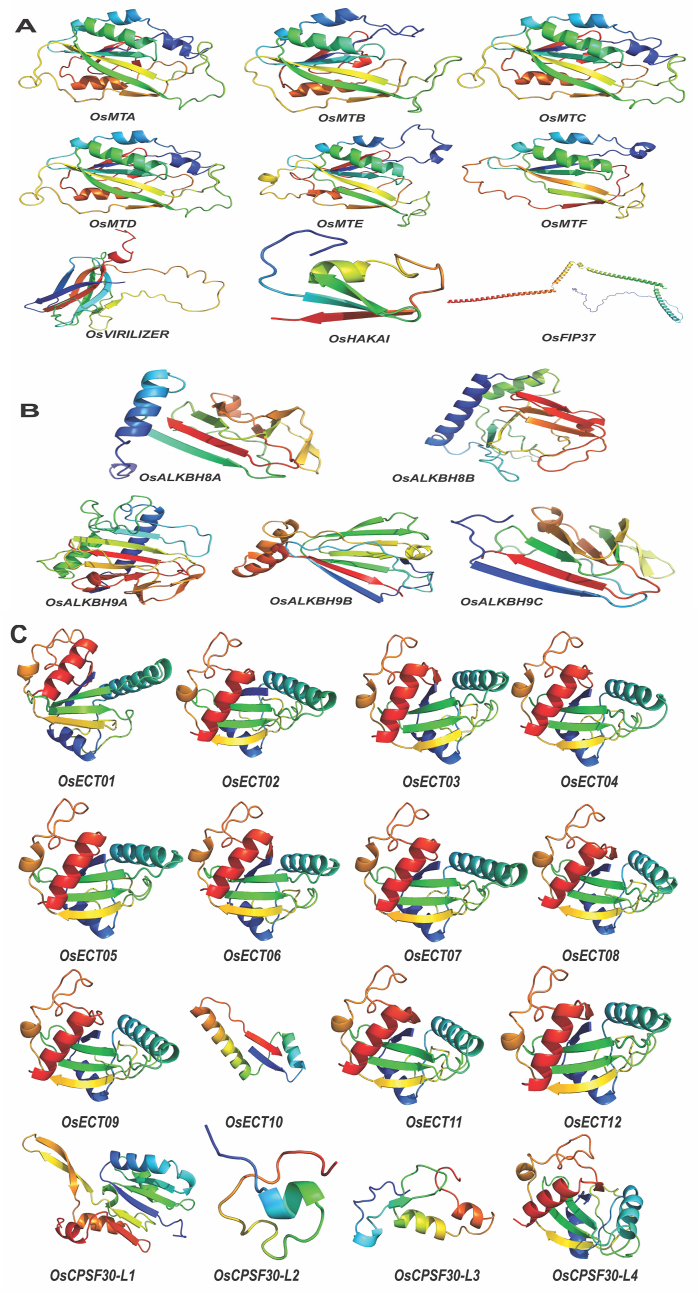
Rice 30 m^6^A proteins 3D structure prediction using AlphaFold2. Domain structures (blue, green, yellow, orange, red, N-terminal to C-terminal) of (A) Writers, (B) Erasers and (C) Readers.

The 3D structure of the 30 m^6^A proteins was predicted using the AlphaFold2 which accurately predicts structure from sequence alignments and provides outputs including 3D coordinates and a confidence metric called pLDDT. pLDDT scores above 90 indicate high accuracy, between 70 and 90 suggest decent backbone prediction, while regions between 50 and 70 should be interpreted cautiously. AlphaFold2 predicts that pLDDT50 regions will appear unstructured and ribbon-like when isolated, yielding loops and domains. Predicted Aligned Error (PAE) is an additional result that gauges confidence in the relative positioning of paired residues. This interactive 2D graph displays the anticipated position error against residue x, aligned with the true structure on residue y. A lower score indicates higher certainty from AlphaFold2 about these residues being closely positioned in a structure. Additionally, AlphaFold2 provides a per-position prediction of LDDT for all five ranked structures and sequence coverage. The 3D structure in PDB format, pLDDT score, PAE score, and sequence coverage of these 30 m^6^A proteins are provided (Fig. S1). The most favourable model out of the five predicted structures was selected to examine structural resemblances among orthologs containing the same domain. While there are variations in the overall protein structure, significant similarities are primarily observed within the domain segments. Within the group of writer proteins, all six protein orthologs containing the MT-A70 domain exhibit an identical domain structure. Conversely, proteins containing the Wtap, VIRN, and HAKAI domains demonstrate distinct domain structures (Fig. 4A). The domain structures of the five-eraser protein orthologs, namely *OsALKBH8A*, *OsALKBH8B*, *OsALKBH9A*, *OsALKBH9B*, and *OsALKBH9C*, exhibit variations (Fig. 4B). Except for OsECT10, all ECT protein orthologs containing the YTH domain exhibit a consistent domain structure. On the other hand, *OsCPSF30-L1* and *OsCPSF30-L4* demonstrate a nearly identical domain structure, which contrasts with the domain structure of *OsCPSF30-L2* and *OsCPSF30-L3* (Fig. 4C). The presence of variations in the domain structure among these reader proteins provides compelling evidence for their wide-ranging functionalities.

### 3.5 Phylogenetic analysis of mRNA writers, erasers, and readers among different species

**Fig. 5:**
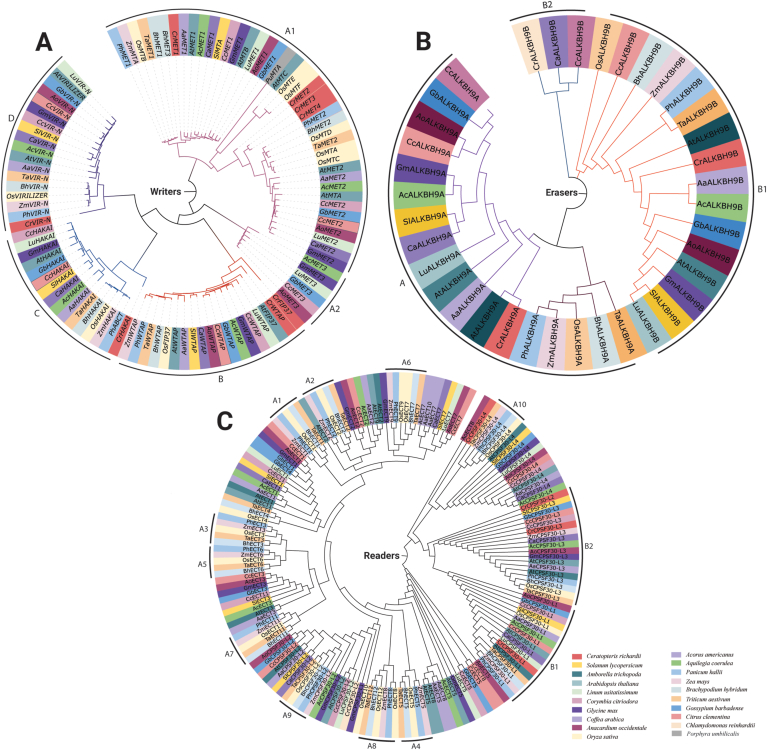
**Evolutionary relationship of m**^**6**^**A regulatory genes among twenty species.** Phylogenetic trees of writers (A), erasers (B), and readers (C), among Rhodophyta, Chlorophyte, Tracheophyte, and Angiosperm (such as eudicot and monocot) were constructed using MEGA X to analyze their evolutionary relationship.

The dynamics of m^6^A were explored in representative species of Rhodophyta, Chlorophyte, Tracheophyte, and Angiosperm (such as eudicot and monocot) using phylogenetic inference, revealing intriguing findings. All the writer orthologs of Methyltransferase, WTAP, HAKAI, and VIRILIZER of selected species form 4 different groups A, B, C, and D (Fig. 5A). *A. coerulea, G. max, L. usitatissimum, G. barbadense, A. occidentale* and *C. citriodora* orthologs of methyltransferases form distinct A2 clad which is out grouped from A1. This phylogenetic tree reveals that OsMTB, OsFIP37, OsVIRILIZER, and OsHAKAI have 4 common closest orthologs of *T. aestivum, B. hybridum, Z. mays*, and *P. hallii*. The most distantly related orthologs of rice writers are from the fern species *C. richardii*. YTH domain-containing eraser protein orthologs form two different groups A and B, where group B has two subgroups B1 and B2 (Fig. 5B). Three different ALKBH9B orthologs of *C. richardii, C. arabica*, and *C. citriodora* form a clad separately. Readers of the different species form clad based on their types. In most cases *A. occidentale* orthologs of readers out grouped from the respective clads. B1 and B2 are the largest clads containing 17 and 18 orthologs, respectively. Group A1-A10 are marked as the same species forming clads for every type of reader (Fig. 5C). Some sister taxa consist of monocot-dicot pairs, while others consist of monocot-monocot and dicot-dicot pairs. For instance, the closely related species *S. lycopersicum* and *C. arabica* share a recent common ancestor and have substantial evidence supporting their nodes. This suggests the possibility that monocot and dicot plants could have diverged from a shared ancestor. These three phylogenetic trees illustrate the conservation of m^6^A dynamics across a diverse range of kingdoms, encompassing plantae, bryophytes, and algae. This indicates that the m^6^A modification is present and functionally relevant in various organisms within these groups.

### 3.6 Expression profiling in different anatomical tissues and developmental stages

**Fig. 6:**
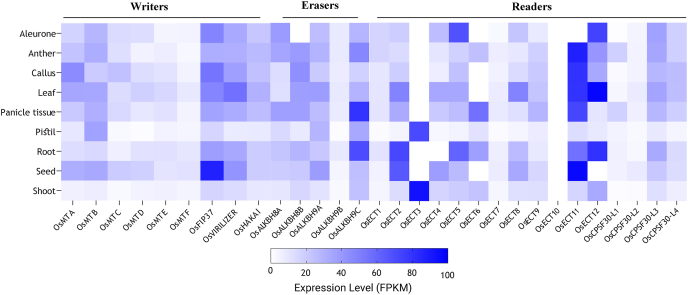
**Expression profile of m**^**6**^**A regulatory genes in different developmental stages and anatomical tissues.** Expression of 9 writer genes, 5 eraser genes, and 16 reader genes (total 30) were analyzed at 9 different tissues such as Anther, Callus, Aleurone, Root, Seed, Panicle, Leaf, Pistil, and Shoot. Rice RNA writer, eraser, and reader gene's genome-wide RNA-Seq data were obtained from the Rice Expression Database and tissue-specific expression levels of the 30 m^6^A genes were carried out to generate a heatmap using the GraphPad prism tool.

Transcript abundance data for all the identified genes were retrieved for 9 anatomical tissues of rice. In various stages of the rice life cycle, different writers, erasers, and reader members displayed varying degrees of expression. No gene was abundantly expressed in all the tissues or even at all developmental stages; instead, most of them displayed expression at medium to low levels. Among all the 9 anatomical tissues of rice such as anther, callus, aleurone, root, seed, panicle, leaf, pistil, and shoot, a medium to a high level of expression was observed predominantly for all 30 rice m^6^A genes (Fig. 6). Few genes showed high expression, while most expressed in a wide range of high to medium and medium to low levels, appearing in a mosaic pattern. Notably, *OsFIP37* is highly expressed in seeds, suggesting it might be involved in seed development. Similarly, *OsECT2* and *OsECT11* also demonstrate significant expression levels in seeds. Moving beyond seeds, *OsECT3* shows preferential expression in shoot and pistil tissues. Meanwhile, *OsECT5* is prominently expressed in root and aleurone tissues. Another gene, *OsALKBH9C*, specifically manifests in root and panicle tissue. The most versatile expression pattern belongs to *OsECT11*, which is found in multiple tissues, including anther, callus, root, seed, panicle, and leaf.

Several genes exhibit lower expression levels across various tissues. Notably, *OsMTD* and *OsALKBH9B* are both expressed at lower levels in the pistil. *OsALKBH8B* shows reduced expression specifically in the aleurone tissue. *OsECT3*, despite its widespread presence, demonstrates lower expression in multiple tissues, including aleurone, anther, callus, root, seed, panicle, and leaf. *OsECT4* is less active in the root. *OsECT6* exhibits diminished expressions in aleurone, callus, root, and seed. *OsECT10* displays lower expression across all tissue types. Lastly, *OsECT11* and *OsECT12* are found to have reduced expression in the aleurone and seed tissues, respectively.

### 3.7 Expression profiling in response to abiotic and biotic stresses

**Fig. 7:**
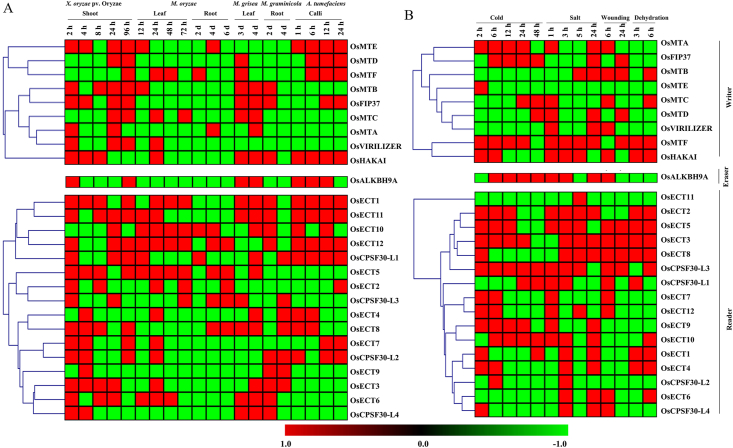
**Expression pattern of m**^**6**^**A regulatory genes under biotic and abiotic stress conditions.** (A) The expression profile was analyzed in response to 5 pathogens: *X. oryzae pv. oryzae, M. oryzae, M. grisea, M. graminicola*, and *A. Tumefaciens*. (B) The expression pattern of 30 genes was analyzed in response to four abiotic stress conditions: cold, salt, wounding, and drought. Fold change in expression as compared to control was used to generate the heatmaps with hierarchical clustering of Manhattan distance correlation in the MeV software package. The colour scale provided at the bottom of the figure represents the level of expression. The stress-induced upregulation and down-regulation of all transcripts are indicated by the green and red colours, respectively.

Fold change in expression data for 26 rice writer, eraser, and reader genes were obtained and evaluated in response to five pathogenic infections: X*. oryzae pv. oryzae, M. oryzae, M. grisea, M. graminicola, A. tumefaciens*, and four abiotic stresses: cold, salt, wounding, and drought at different time points and tissues. In response to the infection of pathogenic organisms, most of the RNA m^6^A components were predominantly upregulated (Fig. 7A). All the rice writers (except *OaHAKAI*) displayed strong upregulation in response to prolonged exposure to *X. oryzae pv. oryzae* for 24–96 h. Only *OsMTC* displays up-regulation after 72 h of *M. oryzae* infection, whilst the other writers showed down-regulation. Infection with *M. grisea* (6 days) and *M. graminicola* (4 days) resulted in widespread downregulation of all writer transcripts. Infection with *A. tumefaciens* (24 h) resulted in the upregulation of five writers including *OsHAKAI*, *OsFIP37*, *OsMTF*, *OsMTD*, and *OsMTE*, the rest of the 4 writers showed downregulation. Erasers expressed sporadically in response to *X. oryzae pv. oryzae*, *OsALKBH9A* showed continuous down-regulation but surprisingly, upregulated at 96 h. Infections with *M. oryzae* for 72 h, *M. grisea* for 6 days, and *M. graminicola* for 4 days resulted in continuous downregulation of *OsALKBH9A*. Infection with *A. tumefaciens* showed continuous upregulation of *OsALKBH9A* during all the time points of 24 h. A cluster of reader genes, namely- *OsECT12*, *OsCPSF30-L1*, *OsECT5*, *OsECT8*, *OsECT7*, *OsCPSF30-L2*, *OsECT1*, and *OsECT11* results in up-regulation after 96 h of exposure to *X. oryzae pv. Oryzae*, whereas *OsECT3* and *OsECT6* showed downregulated by prolonged stress. The rest of the genes remained downregulated in a mosaic pattern. Infections with *M. oryzae* for 72 h result in the upregulation of *OsECT1*, *OsECT10*, *OsECT12*, *OsECT5*, *OsECT2*, and *OsCPSF30-L3* reader genes. On the contrary, only five reader genes including *OsECT12*, *OsECT5*, *OsECT2*, *OsCPSF30-L3*, and *OsECT8* were upregulated in response to *M. grisea* infection. Four reader genes including *OsCPSF30-L4*, *OsECT6*, *OsECT1*, and *OsECT11* were upregulated at the early stage of *M. graminicola* infection but remained downregulated for prolonged stress. Five reader genes cluster including *OsECT12, OsECT5, OsECT2, OsCPSF30-L3*, and *OsECT8* were upregulated constantly during the treatment of *A. tumefaciens*, other five readers including *OsECT12, OsECT5, OsECT2, OsCPSF30-L3*, and *OsECT8* were downregulated.

Abiotic stressors such as cold, salt, wounding, and dehydration caused distinct patterns of change in the expression profile (Fig. 7B). Writer genes such as *OsFIP37, OsMTC*, and *OsMTD* were upregulated at the early stages of cold stress, as did the expression of *OsMTA, OsMTC*, and *OsMTD* after prolonged cold stress. All other m^6^A writers mostly demonstrated downregulation. *OsMTA, OsMTD, OsVIRILIZER*, *OsHAKAI,* and *OsMTF* showed mostly upregulation in an observed period of 1–24h of salt stress. Two writers- *OsMTB* and *OsMTC* showed upregulation only at a late stage and the others remained downregulated at any stage of dehydration stress. Eraser, *OsALKBH9A* showed upregulation against cold and salt stresses but downregulated by wounding and dehydration. Most of the genes are upregulated at the early stage whereas only four genes *OsCPSF30-L3, OsCPSF30-L1, OsECT10*, and *OsECT1* showed upregulation at the late stage of cold stress. Three reader genes- *OsECT6*, *OsCPSF30-L2*, and *OsECT11* showed constant downregulation in cold stress. A cluster of genes including *OsECT5, OsCPSF30-L3, OsCPSF30-L1, OsECT7*, and *OsECT12* showed upregulation at an early stage and downregulation at the late stage of salt stress. A set of reader genes including *OsECT11, OsECT2, OsECT9, OsECT10, OsECT1, OsECT4*, and *OsCPSF30-L2* showed constant downregulation whereas another set of genes including *OsECT5, OsECT3, OsECT8*, and *OsCPSF30-L3* showed constant upregulation throughout the wounding stress. In case of dehydration, a cluster of genes including *OsECT2, OsECT5, OsECT3, OsECT8, OsECT1*, and *OsECT4* showed constant upregulation and another cluster of genes including *OsECT11, OsECT7, OsECT12, OsECT9*, and *OsCPSF30-L2* showed downregulation till 6h of stress. Overall, three readers including *OsCPSF30-L3*, *OsECT3*, and *OsECT5* showed upregulation in all four abiotic stresses at all the observed time points.

### 3.8 Presence of *cis*-regulatory elements in the putative promoter region of m^6^A modulating genes

**Fig. 8:**
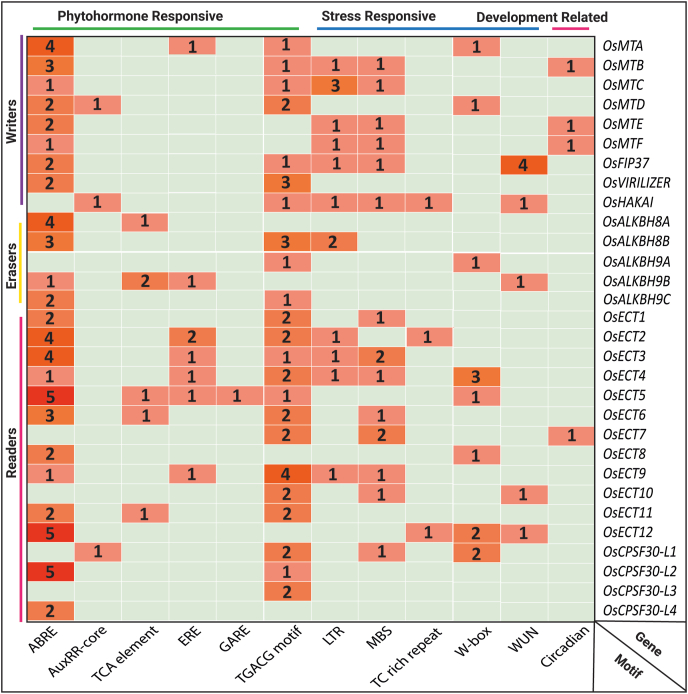
**Cis-regulatory elements of the putative promoter of m**^**6**^**A regulatory genes.** 1000 bp 5′ upstream region of all the identified m^6^A genes were retrieved and analyzed using the PlantCARE database to identify the presence and number of stress-related *cis*-acting regulatory elements. The identified motifs in corresponding genes were represented with different colours.

The presence of several stress-responsive *cis*-elements have been identified, including abscisic acid-responsive element (ABRE), auxin-responsive element (AuxRR-core), fungal elicitor-responsive element (BOX-W1), ethylene-responsive element (ERE), gibberellin responsive element (GARE), heat shock element (HSE), low-temperature responsive element (LTR), MYB binding site (MBS), defence and stress-responsive element (TC rich repeat), wounding and pathogen responsive elements (W box and WUN motif), salicylic acid-responsive element (TCA), element conferring high transcription level (5’ UTR Py-rich stretch) in the putative promoter region of m^6^A modulating genes (Fig. 8). Two writers including *OsHAKAI* and *OsFIP37* have a maximum number of five *cis*-regulatory elements among all the writers. Three different *cis*-regulatory elements were observed in the promoter of three erasers- *OsALKBH8B OsALKBH9A* and *OsALKBH9B* indicating their differential response to different types of stress. In the case of readers, the promoter region of *OsECT5* has the maximum number of 6 *cis*-regulatory elements. The prevalence of these stress-responsive *cis*-elements in the putative promoter region of these genes correlates with the dynamic expression pattern of m^6^A-modulating genes.

## 4 Discussion

Post-transcriptional regulatory markers in the form of more than 150 RNA modifications have been discovered in a wide range of RNA species, including mRNA, tRNA, rRNA, snRNA, and lncRNA [65]. One prevalent post-transcriptional RNA modification is N6-methyladenosine (m^6^A), which is responsible for approximately 80% of all RNA methylation changes in organisms. This affects splicing, export from the nucleus to the cytoplasm, RNA degradation, and translation, among other elements of their lifetime [66,67].

Herein, the presence of 30 m^6^A regulatory genes was identified in the rice genome and correlated with their transcriptional regulation in terms of anatomical distributions, developmental variations, and environmental fluctuations. Previous studies revealed the presence of 29 m^6^A genes in *Arabidopsis* [68], 31 genes in litchi [69], 24 genes in tomato [70], and 34 genes in tea [71]. Genome-wide studies are yet to be performed in other agronomically important plant species to identify the full m^6^A regulatory gene family members. To date, most of the reported plants have exhibited a consistent number of m^6^A regulatory genes, suggesting their stable presence regardless of variations in genomic size.

Exploring the structural characteristics of the identified genes and their associated proteins revealed that domain structure significantly influenced these traits. Gene duplications play a significant role in genetic evolution, with three main evolutionary patterns, which are segmental, tandem, and transpositional replications [72,73]. In our present study, investigating the synteny of rice m^6^A regulatory genes revealed that out of the 30 genes in total, 4 showed evidence of segmental duplication, while 2 genes located on chromosome 12 likely originated from a tandem duplication event. Hence, it seems that the expansion of the rice m^6^A gene family involved contributions from both segmental and tandem duplications. Evolutionary redundancy was demonstrated by segmental duplication in readers and erasers, while evolutionary novelty was indicated by scattered and transposed duplication.

According to the previous reports, the m^6^A methyltransferase complex appears conserved in mammals and plants, with potential divergence in plant m^6^A "writer" complex due to ZC3H13, RBM15, and RBM15B orthologs [74]. Rice, Tomato, and Arabidopsis share similar m^6^A writer components (MTA, MTB, FIP37, VIR, HAKAI), but rice displays another four distinct MT orthologs (*OsMTC, OsMTD, OsMTE,* and *OsMTF*) and domains, suggesting a more intricate m^6^A writer mechanism (Fig. 3B and Table 1).

Within the ALKBH family, *OsALKBH9A* and *OsALKBH9B* were identified as the m^6^A demethylase as well as erasers, impacting fruit ripening by governing the DNA demethylase [75]. Yet, the influence of m^6^A erasers on rice growth, its developmental role, and its potential involvement in fruit ripening through alternate pathways remain unexplored. Interestingly, our evolutionary and structural analyses revealed evident divergence among rice ALKBH family genes (Fig. 3B). Previously, ALKBH proteins, barring ALKBH5 in humans, showed functional variety [76]. For instance, *HsALKBH1* demethylates DNA and RNA, *HsALKBH2* engages in DNA repair, *HsALKBH7* influences fatty acid metabolism and programmed cell death, and *HsALKBH8* is vital for mcm5u biogenesis in tRNA [75]. This evolutionary insight aids the discovery of new m^6^A erasers in rice, while our findings establish a basis for probing the distinct functions of ALKBH members in rice.

In terms of m^6^A reader genes (proteins containing YTH domains), genome-wide identification is limited to a small number of plant species. In rice, 12 such genes have been identified [77], while common wheat, with its larger chromosome count (45 chromosomes), possesses 39 m^6^A reader genes [78], a disparity attributed to its greater chromosome count compared to rice (12 chromosomes). Upon more extensive analysis in our study, the additional understanding of the 30 m^6^A regulatory genes in rice reveals their involvement in alternative splicing mechanisms. Consequently, these 30 genes exhibit the capacity to generate a total of 55 distinct m^6^A regulatory proteins through alternative splicing processes.

The phylogenetic tree analysis unveiled the evolutionary patterns of m^6^A writer, eraser, and reader genes as they evolved across a spectrum of plant species, including Rhodophyta (red algae), Chlorophyte (green algae), Tracheophyte (vascular plants), and Angiosperms (comprising eudicots and monocots). In a prior study, a phylogenetic tree was created by comparing the protein sequences of N6-methyladenosine writers, erasers, and readers in Poplar 84K with their counterparts in Arabidopsis and *O. sativa* to explore their phylogenetic relationships [79]. In another study on m^6^A genes of Arabidopsis, it was found that methyltransferase domains from various species share close relationships, while other components of methylation are organized differently. Notably, human writers MET, ALKBH, and YTH do not closely resemble their counterparts in other eukaryotes [68]. This study shows some sister taxa involve monocot-dicot, others monocot-monocot, and dicot-dicot pairs. E.g., *S. lycopersicum, C. arabica* share a recent ancestor, implying shared monocot-dicot divergence. These phylogenies highlight m^6^A conservation across Plantae, bryophytes, and algae, indicating its functional relevance across diverse organisms (Fig. 5). Moreover, the presence of various domains among m^6^A writer, eraser, and reader members in diverse clusters highlights the range of functions these members possess in plants. These proteins likely evolved to offer different protective roles against a variety of stresses. 3D structure prediction supports the idea that homologous proteins with shared domains display similarities in their domain structures. Moreover, the unique structure of the writer, reader, and eraser proteins, along with their domains, emphasize their diverse functions. Consequently, precise identification and in-depth exploration of this gene diversity become essential.

It is not apparent how exactly m^6^A modification controls the fate of RNA molecules in plants. However, research has demonstrated that modifications to any part of the m^6^A system whether it be the writer, eraser, or reader may cause regulatory system abnormalities, which in turn can cause irregular growth and development. Previous studies revealed that in rice and Arabidopsis total m^6^A levels drop dramatically in response to the loss or reduction of m^6^A writers such as MTA, MTB, VIRILIZER, and FIP37 [[80], [81], [82]]*.* Variable phenotypes are brought on by the lack of m^6^A alteration, including embryonic mortality, epidermal hair formation anomalies, faulty leaf sprouting, and excessive proliferation of vegetative shoot apical meristem [[81], [82], [83], [84]]. In this study, a mosaic pattern of medium to high expression is observed among the 30 rice m^6^A genes across the 9 anatomical tissues, with notable high expression of *OsFIP37*, *OsECT2*, and *OsECT11* in seeds, *OsECT3* in shoot and pistil, *OsECT5* in root and aleurone, and *OsALKBH9C* in root and panicle; *OsECT11* exhibits the most versatile expression across anther, callus, root, seed, panicle, and leaf, while several genes, including *OsMTD*, *OsALKBH9B, OsALKBH8B, OsECT3, OsECT4, OsECT6, OsECT10, OsECT11*, and *OsECT12,* display lower expression levels in various tissues, such as pistil, aleurone, and root (Fig. 6). The *MTA* mutant seeds with a low level of m^6^A modification display a halted developmental progression at the globular stage. FIP37's involvement in m^6^A-mediated mRNA modification is crucial for balancing stem cell proliferation and organ development in Arabidopsis [85]. It highlights the intricate regulatory mechanisms underlying plant growth and adaptation. A previous study reveals that ALKBH10B specifically regulates the floral transition process by demethylating transcripts of key flowering time genes, including flowering locus T (FT) and squamosa promoter binding protein-like 3 (SPL3) in Arabidopsis [86]. Another study focuses that YTH proteins are involved in various plant processes, including embryogenesis, flowering transition, root development, stem cell fate determination, circadian rhythm, leaf morphology, leaf coat development, nitrate signalling, fruit maturation, gametophyte development, phytohormone responses, and stress responses [87]. The expression pattern of m^6^A genes observed in this study provides clear evidence of their involvement in rice growth and development.

Inefficient decay of senescence-related transcripts (e.g., ORESARA1, SAG21, NAP, and NYE1) in MTA leads to accelerated senescence during DILS. A study suggests that m^6^A modification plays a role in regulating plant senescence, offering potential targets for enhancing stress tolerance in crops [88]. In plants, m^6^A modification system is very sensitive and works in intricate ways in response to heat, salt, and drought stress, demonstrating its essential function in stress tolerance processes. Arabidopsis salt tolerance is regulated in part by m^6^A alteration, which increases the stability of transcripts under salt stress [83]. Maize's expression levels of writer and reader proteins rise during drought stress, but m^6^A modification declines, suggesting a role for these proteins in drought resistance. Various maize genotypes have varied m^6^A mutations, indicating different regulatory mechanisms for drought resistance [89]. Heat stress triggers a response in the Arabidopsis reader protein ECT2, which moves to stress granules in the cell. This may influence the location of mRNA [90,91]. Research has shown that reader proteins are essential for short-term stress responses because they modify RNA more directly and quickly than writers and erasers. Some writers, such as *OsMTA* and *OsHAKAI*, exhibited acute downregulation in response to specific stressors and time durations, while others exhibited upregulation. Erasers, such as *OsALKBH9A*, exhibited both a fluctuating pattern in response to the stressor and the duration. Some reader genes, such as *OsECT12, OsCPSF30-L1, OsECT5, OsECT8, OsECT7, OsCPSF30-L2, OsECT1*, and *OsECT11*, displayed upregulation in response to specific stressors and time points, whereas others displayed downregulation. During biotic stress in rice plants, complex and context-dependent alterations in gene expression are observed.

In contrast to a previous study on rice, which showed increased *OsFIP* levels under cold stress and decreased levels of *OsMTA, OsMTB*, and *OsVIRILIZER* under cold, drought, or salt stress [92], the current study observed upregulation of writer genes like *OsFIP37, OsMTC*, and *OsMTD* under cold stress. Additionally, under salt stress, *OsMTA, OsMTD, OsVIRILIZER*, and *OsMTF* were upregulated, and under dehydration stress, *OsMTF* and *OsHAKAI* consistently exhibited upregulation (Fig. 7B). In the previous study, it was found that levels of ALKBH1 in rice increased significantly upon drought, cold, or ABA treatment, while ALKBH6, ALKBH8B, and ALKBH10A showed decreased expression under drought, ABA, or cold conditions [92]. Among the YTHDs in rice, responses to different abiotic stresses varied; for instance, YTHD05, YTHD06, YTHD07, and YTHD09 were downregulated by cold stress, while YTHD03 and YTHD08 increased under submergence and heat stress. Notably, none of these YTHDs exhibited changed expression under salt stress, and YTHD01, YTHD02, YTHD03, YTHD04, and YTHD08 did not respond to cold stress [77]. In this study, *OsALKBH9A*, an eraser gene, displayed varying expressions in reaction to different stresses. Similarly, reader genes showed distinct expression patterns under stress conditions. The dynamic expression pattern of m^6^A writer, reader, and eraser elements in both regular and stressful circumstances indicates the essential function of m^6^A methylation in plant growth and the reaction to stress. Moreover, CREs, which encompass noncoding DNA with binding sites for transcription factors and regulatory molecules, impact transcription, guiding plant growth, development, cell differentiation, and stress responses [93]. The potential promoter sequences of 30 m^6^A genes contain different CREs that could potentially react to phytohormones, signals related to plant development, as well as biotic and abiotic stress (Fig. 8).

Collectively, m^6^A regulatory genes display distinctive expression patterns under various stress treatments, possibly because of the influence of promoter motifs on their expression. Further biochemical characterization is necessary to fully understand the dynamic functions of all three categories of m^6^A modifiers (writers, erasers, and readers) in stress regulation. This offers the opportunity to fine-tune gene expression and modify the plant epitranscriptomic for desired features.

## 5 Conclusion

We have conducted a genome-wide search for m^6^A regulatory genes in rice. The identification of genes, their chromosomal position, duplication analysis, phylogenic connection, and duplication analysis as well as the existence of *cis*-regulatory elements have been thoroughly analyzed. The expression of regulatory RNA methylation members in distinct plant tissues at different developmental phases and stress conditions have also been profiled Their sharp fluctuations of expression in response to stresses have suggested a significant role in the stress modulation pathways of rice, which paves the way for further molecular and functional characterization to elucidate the detail workings in this regard.

## Funding

AG has received partial funding from the 10.13039/501100007944Shahjalal University of Science and Technology Research Center (LS/2023/1/01) and the Ministry of Education, Government of the People's Republic of Bangladesh (LS20201353) to conduct the research.

## CRediT authorship contribution statement

**Mahbub Hasan:** Writing – original draft, Visualization, Methodology, Investigation. **Zakia Sultana Nishat:** Methodology, Investigation, Formal analysis, Data curation. **Md. Soyib Hasan:** Methodology, Investigation, Data curation. **Tanvir Hossain:** Validation, Supervision, Software, Conceptualization. **Ajit Ghosh:** Writing – review & editing, Funding acquisition, Data curation, Conceptualization.

## Declaration of competing interest

The authors declare that they have no known competing financial interests or personal relationships that could have appeared to influence the work reported in this paper.

## Figures and Tables

**figs1:**
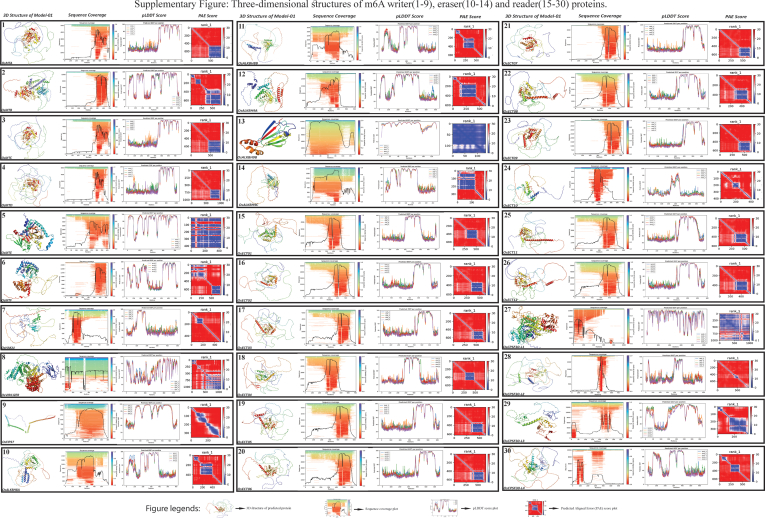


## Data Availability

Data will be made available on request.
